# What Need for Speed? Lizards from Islands Missing Predators Sprint Slower

**DOI:** 10.3390/ani15182651

**Published:** 2025-09-10

**Authors:** Sarah L. Semegen, Johanna L. Fornberg, Peter A. Bednekoff, Johannes Foufopoulos

**Affiliations:** 1 School for Environment and Sustainability, University of Michigan, 440 Church St., Ann Arbor, MI 48109, USA; 2 Biology Department, Eastern Michigan University, 441 Mark Jefferson Science Complex, Ypsilanti, MI 48197, USA

**Keywords:** ecology, predation pressure, locomotor performance

## Abstract

Island taxa are frequently susceptible to introduced predators. This susceptibility is thought to be caused by the loss of key ancestral antipredator physiological adaptations during long periods of evolution under reduced predation pressures. Here, we test the hypothesis that island species have reduced locomotor abilities. While locomotor abilities are critical for escaping predation, little is known on how the presence of different types of native predators influences these abilities by maintaining selective pressure. To fill this gap, we documented sprint speed in the Aegean wall lizard (*Podarcis erhardii*) from the Aegean islands (Greece) with varying levels of predation pressure. We show that lizards from islands where mammalian predators were present sprinted fastest. Lizards sprinted at an intermediate speed where predators other than mammals were present, and lizards sprinted slowest on islands where no predators were present. Longer periods of evolutionary isolation in island environments were also associated with diminished sprinting speeds. These results indicate that lizards from the lowest-predation islands are the most vulnerable and preventing the introduction of invasive predators should be prioritized for these island systems.

## Simple Summary

Island species have evolved in an environment of reduced threats, including diminished predation pressures, and are predicted to have down-regulated physiological defenses against such threats. We measured one of these defenses, escape running speed, for lizards from 22 Mediterranean islands. We find that this critical antipredator defense is reduced in lizards from islands with fewer types of predators. While predator species number on an island affects sprint speed for that population, not all predator types are equally important. Presence of efficient mammalian predators like cats and stone martens is particularly influential in the retention of high sprinting speeds in their lizard prey. Overall, our approach allows us to predict the running ability of an island population based on the types of predators on that island. This is important because reduced running ability correlates with extinction risk by new invasive predators. Our approach therefore helps predict population vulnerability and can help guide investment of scarce conservation funds for maximum effect.

## 1. Introduction

More than 60% of recent recorded extinctions are from islands [1], driven in part by the introduction of exotic predators to which island taxa are frequently particularly susceptible. Appropriate antipredator behaviors are critical to survival, and are often costly [2,3]. For example, retreating into a refuge disrupts thermoregulation and feeding by lizards [4,5]. Because antipredator defenses incur costs, especially if deployed accidently, the degree of their expression is expected to reflect predation risk in a local area [3,6,7].

Island ecosystems generally have fewer species and often are missing types of predators found in mainland ecosystems. Organisms from islands isolated over very long periods of time may have obviously reduced defense mechanisms, leading to “island tameness” [2,3,6,8,9,10,11,12,13]. This lack of defenses makes island species particularly vulnerable to introduced novel predators [2,10]. Indeed, colonization and invasion by novel predators has led to dramatic reductions and even extinctions in native species populations on islands worldwide [1,14,15,16]. Worldwide, feral cats alone are responsible for at least 48 documented extinctions of island species [17].

The ability to flee from an approaching predator is a key antipredator defense and is thought to have physiological costs including acquisition and maintenance of muscles [18,19,20,21]. Previous studies of locomotion in lizards have focused on sprint speed as a measure of fitness for ecomorphs focusing mainly on their ability to escape quickly in distinct habitats [22]. Some studies have shown that elevated sprint speed is associated with reproductive success [20,23] and survivorship in lizards [19]. Previous studies comparing one island to one mainland population lizards suggest locomotory performance can decline quickly with reduced predation pressure [24,25]. Other studies show that sprint speed in lizards can change with body temperature [26] and is influenced by body size (SVL), leg length [21,27], tail autotomy [28], movement patterns [29,30,31], and motivation [17,32].

We studied sprint speed in Aegean wall lizards (*Podarcis erhardii*, Bedriaga, 1886), a widespread species that occurs in numerous distinct populations throughout the Cyclades archipelago in the Aegean Sea, Greece. Varying degrees of predation pressure on the Cyclades islands provide an ideal environment to study the influence of predation on antipredator behaviors [33]. Previous studies in this region demonstrated that tail autotomy and flight initiation distance were less developed on islands with fewer types of predators, and more developed on islands with more types of predators [3]. The goals of this study were to: 1. Quantify how sprint speed varies among different island and mainland populations in response to differing predation environments. 2. Determine which specific factors drive the evolution of sprint speed.

## 2. Materials and Methods

### 2.1. Study System

The Cyclades archipelago is a cluster of 250+ mostly land-bridge islands located in the central Aegean Sea (Greece). Since the end of the last glacial maximum approximately 18,000 years ago, rising sea levels have fragmented former mainland coastal regions. As Holocene sea level rise separated the area, populations of plants and animals also became increasingly isolated, thus setting the stage for the evolution of locally adapted island phenotypes [33,34,35].

The study area has long, warm, dry summers and mild, rainy winters as is typical of Mediterranean region climates [35]. Humans have inhabited the region for thousands of years, altering the vegetation through agriculture and animal grazing. Today, island habitats consist typically of agricultural fields edged by dry-stone walls and embedded in a matrix of spiny, summer-deciduous, low-growing woody vegetation known as *phrygana* [36].

The Aegean wall lizard (*Podarcis erhardii*, Lacertidae) is a medium-sized lizard, typically measuring between 48 and 78 mm from snout to vent [3,37,38]. *P. erhardii* is found throughout the southern Balkans, mainland Greece, and many Aegean islands, and is common throughout the Cyclades island group [37]. The species has presently over 26 different subspecies described, though the validity of many is still under discussion (37). The species has broad habitat preferences, although it is not found in closed-canopy forests. It can be found in particularly high densities in areas where appropriate refugia, like dry-stone walls, are present. The diet of *P. erhardii* consists predominately of arthropods, although it occasionally includes vegetation and fruit [37]. Aegean wall lizards are poor overwater dispersers. While dispersal by humans is suspected elsewhere [39], genetic studies of our study populations confirm that each population has evolved in response to locally prevailing conditions since isolated by rising sea levels [35,40,41,42]. Many island subspecies have been proposed, reflecting aspects of the broad morphological and genetic variation found in the different Aegean islands and pronounced adaptation to local environments [42,43].

### 2.2. Island Characteristics

**Figure 1:**
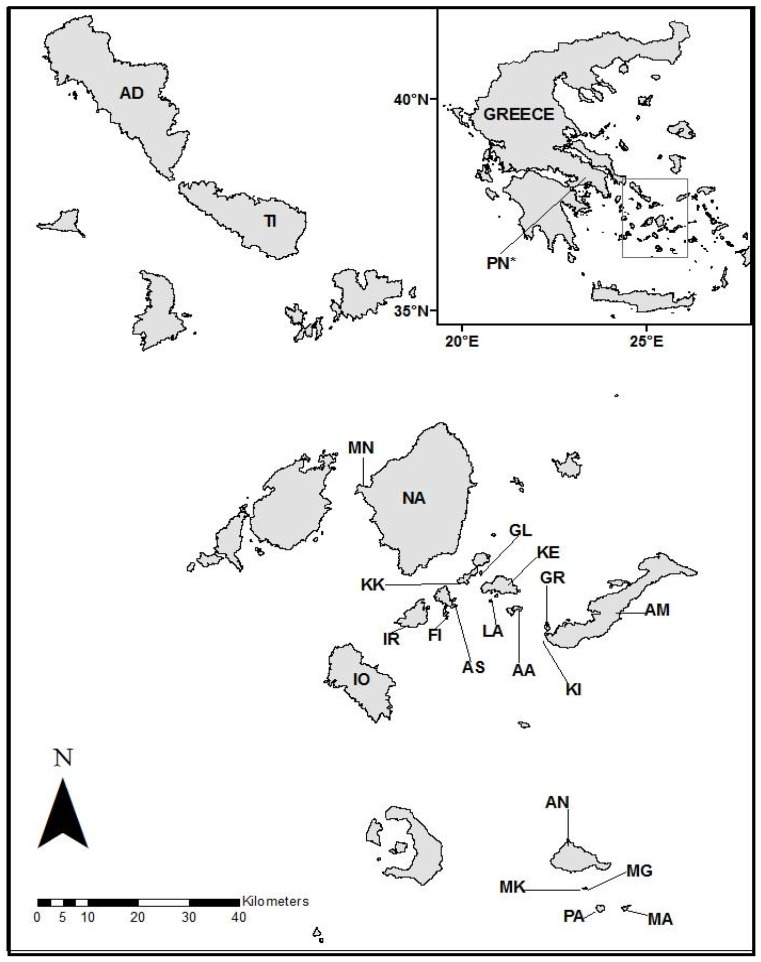
Map of Greece and of study sites in the Aegean Sea. Amorgos (AM), Anafi (AN), Andros (AD), Ano Antikeros (AA), Aspronissi (AS), Fidussa (FI), Glaronissi (GL), Gramvoussa (GR), Ios (IO), Iraklia (IR), Kato Koufonissi (KK), Keros (KE), Kisiri (KI), Lazaros (LA), Makria (MA), Mando (MN), Megalo Fteno (MG), Mikro Fteno (MK), Naxos (NA), Pachia (PA), Tinos (TI), Parnitha (Mainland) (PN*). * The mainland location is shown on the inset map.

Study sites (*n* = 22 see Figure 1) were selected to include a range of island sizes, ages (i.e., periods of isolation), and also predation environments. Using methods described more fully in Brock et al. [3] and Pafilis et al. [33], we assessed predator presence on an island by combining published information with field surveys for any direct (live or dead individuals) or indirect (burrows, fecal matter, tracks) evidence of predators. Predator species were grouped into six types: rats (R), predatory mammals (M), raptorial birds (B), vipers (V), sand boas (SB), and other snakes (OS). Each predator type is characterized by distinct hunting strategies, which likely results in different antipredator behaviors [3]. Rats (*Rattus rattus*), for example, are considered in a category distinct from “mammals” because in the Cyclades the rats are small, opportunistic predators that do not prey on lizards as effectively as feral cats (*Felis catus*) and stone martens (*Martes foina*) [3] which use burst speed to capture prey [44]. Snakes are separated into three types based on hunting strategies. Vipers (*Vipera ammodytes*) are sit-and-wait predators that ambush and envenomate prey [33]. Sand boas (*Eryx jaculus*) are constrictors that will prey on adult and juvenile lizards but mostly feed on lizard eggs [3]. ‘Other snakes’ include several diurnal colubrid taxa (e.g., *Natrix natrix, Elaphe quatuorlineata, Dolichophis caspius*) that actively search for prey [33].

Lizard population density measurements were taken along a 100 m long, 5 m wide transect within each study site. Following established protocols [3], lizards seen or heard were counted by an observer (SS) while walking along the transect during peak hours of lizard activity (09:00–11:00, and 15:00–17:00) and under optimal environmental conditions (20–25 °C; sunny; wind speeds < 2 Bf). Previous research has suggested that habitat openness is an important determinant of sprint speed [45,46]. At each location, we measured habitat openness along a 100 m long transect randomly laid out within the habitat occupied by *P. erhardii* at that location. Substrate type, vegetation type, and height above the ground were recorded at 1 m intervals along the transect. Areas with vegetation < 5 cm high were categorized as open habitat amenable to rapid running.

### 2.3. Animal Methods and Morphology

Lizards were caught by lassoing or baiting with mealworms (*Tenebrio* sp. larvae) during early summer (May–early July) 2016. A sample of 18–34 lizards were taken from each location. Each lizard was given a unique, temporary number on the back using a non-toxic marker. While in captivity, lizards were held in 60 cm × 41.6 cm × 33.7 cm terraria, were provided with water ad libitum, and fed mealworms once a day. Morphometric data, including snout to vent length (SVL), hindlimb length, and hindleg span (hindspan) were measured using digital slide calipers [47,48]. Lizards were sexed and body mass measurements were collected using a spring-loaded scale, (Pesola model 10020). Lizards were returned to the place of capture after all morphological and performance measurements were recorded.

### 2.4. Sprint Trials

Sprint speed was measured using a 230 cm long and 40 cm wide wooden racetrack. Every lizard ran 3 sprint trials to ensure that there was at least 1 trial in which that lizard sprinted as fast as it could. Before each sprint trial, lizards were allowed to thermoregulate for at least an hour in a temperature gradient. Sprinting was induced by a combination of sudden sound and a looming object. Specifically, we clapped our hands then thrust a soft-bristled brush toward the lizard from behind. Temperatures were taken by cloacal thermometer immediately after each trial. Trials were spaced at least 1 h apart to allow the lizards time to recover.

Using a video camera (GoPro, San Mateo, CA, USA; Hero Black 4; 1280 × 720 px), video of each trial was recorded at 240 frames per second; the camera was positioned 1.5 m above the racetrack, so that a clear dorsal view of the running lizard was visible for at least one full meter of the racetrack. Peak velocity in each trial was measured using the video analysis tool SAVRA (code: https://github.com/bkazez/savra, accessed on 1 April 2016) custom-built for Donihue [48]. For the first 1 m of lizard running in each video, a measurement of the distance traveled between every 5-frame sequence was calculated, and fit with a quintic spline using the SPAPI function in MatLab (MathWorks Inc., Natrick, MA, USA) as described in Donihue [48] in order to calculate velocity of the lizard in m/sec. The fastest of the three trials for each lizard was selected for use in analyses. Gravid females were excluded from the analysis, as were trials where individual lizards did not run normally.

### 2.5. Statistical Analysis

Sprint speed, most morphological values (SVL, hindlimb length, and hindspan length), and island characteristics (island age, island area, and lizard density) were Log_10_-transformed to ensure normality of residuals. Visual inspection and heterogeneity tests confirmed that error variation was approximately Gaussian and uniformly distributed in all analyses.

Pearson correlations were used to explore relationships between sprint speed and morphological characteristics, as well as island characteristics (island age, island area, % open habitat, lizard density, and number of predator types).

We used a linear mixed-model approach and investigated how sprint speed was influenced by different ecological drivers by comparing models that contained different predation variables. All models included sex, lizard body size (snout–vent length), and lizard body temperature to control for factors that often affect lizard running performance. Snout–vent length is typically used as a measure of body size, which is linked to stride length and sprint speed, and body temperature has also been broadly linked to sprint speed [18,22,26,30,49,50]. Our aim in including these three factors in all models was to remove potential confounding effects so we could focus on effects of predators.

We compared 8 a priori models to identify which predator types were most important in shaping sprint speed. Models looked at the presence or absence of any predators, the number of types of predators, and presence of individual predator types. The binary “Zero Predation” model separated islands with no predators present from islands with any type of predator. The “Sum of All Fears” model counted the number of predator types found on each island, assuming each predator type added equal predation pressure to the system, as described in Brock et al. [3]. Alternatively, another model estimated the effect of each predator type individually and simultaneously (R+SB+V+B+OS+M). This model considers the fact that predator types are not the same and each might affect sprint speed in a unique way. Based on a priori knowledge, the other five models emphasized mammals (M) and predatory birds (B) as the predators most likely to affect locomotor performance. In order to catch prey, mammals stalk and chase prey, often capturing an animal after a burst of speed [17,44]. Predatory birds utilize a similar approach: after locating prey from above they swoop down to capture an animal [51]. Statistical models of birds, mammals, as well as birds and mammals combined, were calculated with and without separating the zero predation islands. Including zero predation has the effect of calculating whether other predators beyond mammals and/or birds had an impact on sprint speed.

Previous research in this study system suggested island age was important to other antipredator defenses [3], so island age (Log_10_-transformed) was added as another fixed effect to the 8 models stated above. Models were then compared using Akaike Information Criteria, using AICc values and the associated Akaike weights [52].

## 3. Results

### 3.1. Variation and Simple Correlations

Sprint speed varied widely between individual lizards and between islands (range: 1.04–4.37 m/s, $\bar{x}$ = 1.75 ± 0.017 m/s, *n* = 526, Table 1). Sprint speed was positively correlated with lizard body temperature and positively, yet weakly, correlated with lizard SVL (Table 2). Across islands, sprint speed was negatively correlated with lizard density and island age, and positively correlated with number of predator types and with island area (Table 2).

### 3.2. Model Selection

**Figure 2:**
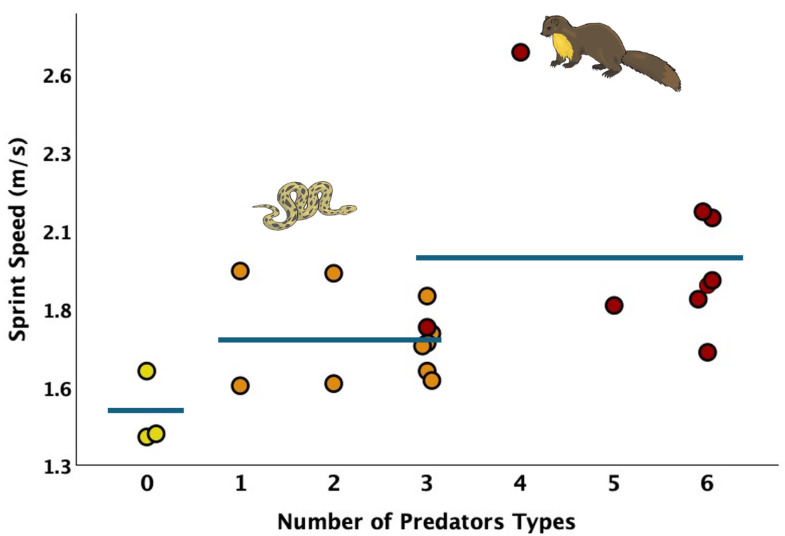
Scatterplot of island sprint speed versus number of predator types found on each island. Islands without predators (light shading) had the slowest sprint speeds. Islands with some predators present (intermediate shading) had intermediate sprint speeds. Islands with the highest sprint speeds were islands with mammalian predators present (dark shading). The three horizontal blue lines present averages for these three categories. Mammalian predators were stone martens and house cats. They were found on one island with three types of predators and all islands with four, five, or six types of predators. Other predators include rats, predatory birds, and types of snakes. In this study, such predators occur without stone martens or house cats on islands with one or two types of predators, and all but one of the islands with three types of predators. Details on the types of predators found on each island can be found in Table 1. Maximum sprint speed across three trials was measured for each lizard. Each dot shows the average of these measurements for all lizards from an island.

Among our models, sprint speed was best explained by the presence of mammalian predators on islands, yet the second- and third-best models suggest that islands without predators also differ from islands with some predators (Table 3). Overall lizards on islands with mammals ran an average of 32% faster than lizards on islands without predators; lizards ran at intermediate speeds on islands with other predators but not mammals present (Figure 2, see also Table 1).

**Figure 3:**
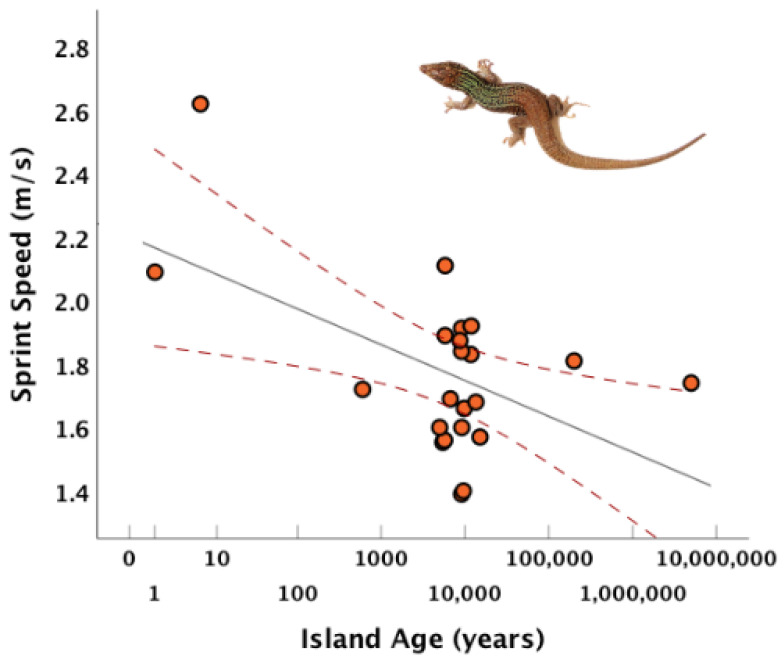
There is a negative correlation between island sprint speed and time of isolation. The highest sprint speeds are found on the youngest sites and decline with increasing island age. Scatterplot of island sprint speed versus time of island isolation (Log_10_-transformed) (r = −0.494, *p* = 0.019, *n* = 22). Gray line shows best fit line. Red dotted lines indicate the 95% confidence interval around the best fit line. Maximum sprint speed across three trials was measured for each lizard. Each dot shows the average of these measurements for all lizards from an island.

In contrast to previous studies, knowing the overall number of predator types (“Sum of All Fears”) was not very predictive. Estimating specific effects for each predator type (“R+SB+B+V+OS+M”) led to our worst performing models. While lizards from older islands sprinted more slowly (Figure 3), adding island age to a model generally decreased how the model performed (Table 3).

## 4. Discussion

This study measured sprint speed in lizards from islands with different predator assemblages. Lizards sprint fastest from islands where mammals are present, and slowest from islands where no predators are present (Figure 2). The main mammalian species on the islands, feral cats (*Felis catus*) and stone martens (*Martes foina*), capture lizards by stalking and then chasing them. Accordingly, the ability to produce fast, anaerobically powered bursts can help lizards avoid capture by these predators [53]. Lizards from islands without mammalian predators but with other predator types present sprinted at intermediate speeds. This result indicates that the ability to produce rapid sprint bursts is maintained when at least one predator type is present (Figure 2). Hence, the presence of any type of predator effectively “keeps the lizards on their toes”. This result is consistent with previous research which showed Tammar wallabies maintain antipredator behavior when a single predator species is present [6].

Because of the underwater geomorphology of the region, most of the study islands became isolated in the period between 1000 and 10,000 years ago. One exception is Mando that became separated from larger, predator-rich Naxos in the decade before this study. Mammalian predators persist on Mando and lizards from this island show exceptionally high running speeds. Their high speeds are perhaps also the result of an unusual sandy substrate reducing the risk of high-speed collisions with rocks. Although lizards from longer-isolated islands sprinted significantly more slowly (Table 2, Figure 3), adding time of isolation to predator diversity models did not further explain sprint speed values (Table 3). This suggests that duration of isolation appears to be important primarily by leading to the progressive loss of predators from islands. Indeed, history of isolation is known to predict overall species loss for these islands [34]. Nonetheless, knowing current predation risk is sufficient to explain the results reported in this paper.

In the absence of predators, we expect that energetically costly antipredator behaviors are lost over time [2]. Adaptive and non-adaptive contributions to this loss may need very different amounts of time. For example, given that maintenance of the muscles required to achieve high speeds is energetically costly, it is likely that once predators disappear, these muscles will be lost over evolutionary time. Vervust et al. [25] suggest that changes in responses to predators, such as flight initiation distance and speed, can happen in a matter of decades. By this standard, adaptive reductions in antipredator behaviors had ample time to act on all but the youngest island in our sample. On the other hand, locomotor performance may decline on small, old islands for reasons besides any adaptive response to reduced predation risk. Small, long-isolated populations lose genetic diversity, and inbreeding depression is known to undermine a variety of physiological processes including locomotor performance [53,54,55]. Nonadaptive loss of antipredator abilities is likely to occur over a far longer period than adaptive reduction in costly antipredator behaviors.

In our study system, many factors correlate with island size [56]. In particular predator community richness increases with island size, and mammalian predators are likely only on the largest islands. Larger islands will also tend to have larger populations of lizards and be less affected by genetic drift. Therefore, non-adaptive loss of antipredator abilities is likely to depend on island size and effective population size in combination with period of isolation.

In our system, we only could study three islands where predators are absent, and our results suggest they are of exceptional scientific and conservation value. Lizards from these islands displayed unique behaviors and an apparent lack of fear when captured and handled for research [3,13]. Such tameness is common in island species, especially species that have been isolated for millions of years, such as on the Galapagos islands [2,10,57]. Research with other *Podarcis* species suggests that antipredator adaptations can be lost relatively quickly and once lost they may be lost permanently [11,13,53]. In Galapagos’ marine iguanas, recent experience with introduced predators leads to increased flight distances, though this has not been enough to reduce predation by invasives [10,57]. Inability to adapt swiftly to increased predation and novel predators is thought to have led to a loss of island species across the planet [1,14] and sprint speed is considered to be a trait of conservation importance in island lizards [58]. Our research suggests that island species without a historic presence of mammalian predators in general, and species from islands without any predators at all should be considered highly vulnerable to exotic predators. These criteria can be used to predict endangerment risk across different populations and prioritize scarce conservation funds.

## 5. Conclusions

Aegean wall lizards have reduced sprint speed on islands without mammalian predators and further reduced sprint speed on islands without any predators at all. These results highlight the importance of mammalian predators to selection for sprint speed, and the exceptional vulnerability of island populations without predators to the introduction of mammalian predators such as house cats.

## Figures and Tables

**Table 1:** Summary table. Island and lizard population characteristics. Islands are listed first by increasing number of types of predators, then by increasing sprint speed. Predator types for each island are listed (r = rats [*Rattus rattus*], sb = sand boas [*Eryx jaculus*], b = predatory birds, v = vipers [*Vipera ammodytes*], os = other saurophagous Colubrid snakes, M = mammals). Sprint speed is given in meters per second. The isolation period is given in years. Island area is given in square kilometers. Morphological characteristics of body size (SVL) and hindspan are given in mm. Percent openness is the percent of open habitat per 100 m transect. Lizard density is the number of individual lizards seen along a 100 m × 5 m transect.

Island	Number of Types of Predators	Types of Predators Present	Mean Sprint Speed (m/s)	Isolation Period (Years)	Island Area (km^2^)	Mean SVL (mm)	Mean Hindspan (mm)	Percent Openness	Lizard Density Ind/100 m
Lazaros (LA)	0	None	1.39	9100	0.01	68.75	73.43	61	13
Megalo Fteno (MG)	0	None	1.40	9580	0.06	59.91	62.13	57	20
Mikro Fteno (MK)	0	None	1.60	5000	0.03	54.12	59.41	70	4
Aspronissi (AS)	1	r	1.55	5450	0.04	60.84	66.41	68	8
Pachia (PA)	1	r	1.92	11,850	1.36	55.92	58.71	61	7
Kisiri (KI)	2	r, b	1.56	5700	0.01	56.31	61.69	83	2
Glaronissi (GL)	2	r, b	1.91	5650	0.16	60.41	65.23	88	3
Ano Antikeros (AA)	3	r, sb, b	1.57	15,150	1.05	59.16	62.50	61	1
Keros (KE)	3	r, sb, b	1.60	9150	15.05	56.52	60.45	47	5
Makria (MA)	3	r, sb, b	1.68	13,500	0.5	56.49	63.22	67	5
Gramvoussa (GR)	3	r, sb, b	1.69	6700	0.76	59.60	63.90	60	1
Fidussa (FI)	3	r, sb, b	1.72	600	0.63	60.01	65.83	77	6
Anafi (AN)	3	r, b, M	1.74	3,600,000	49	57.30	61.99	85	4
Kato Koufonissi (KK)	3	r, sb, b	1.84	9100	4.30	58.46	67.17	67	5
Mando (MN)	4	r, sb, b, M	2.62	7	0.025	62.53	70.41	71	2
Amorgos (AM)	5	r, sb, b, os, M	1.81	200,000	123	61.91	69.63	47	4
Iraklia (IR)	6	r, sb, v, b, os, M	1.66	9800	18.08	59.40	63.71	70	4
Ios (IO)	6	r, sb, v, b, os, M	1.83	11,750	109.03	64.40	71.61	80	2
Naxos (NA)	6	r, sb, v, b, os, M	1.87	8700	448	60.87	69.22	74	6
Andros (AD)	6	r, sb, v, b, os, M	1.89	5800	379.95	68.49	77.60	71	2
Parnitha (PN*) (* Mainland)	6	r, sb, v, b, os, M	2.09	0	1000	65.22	82.86	67	4
Tinos (TI)	6	r, sb, v, b, os, M	2.11	5800	194.5	67.36	77.69	87	1

**Table 2:** Results of simple correlations between sprint speed and morphological measures of each lizard and characteristics of each island.

	Sprint Speed (Log_10_-Transformed)		
Morphology	Pearson r	*p*-Value	n
Body Temperature	0.239	<0.0001	526
SVL (Log_10_-transformed)	0.087	0.045	526
Hindlimb (Log_10_-transformed)	0.234	<0.0001	519
Hindspan (Log_10_-transformed)	0.195	<0.0001	526
Island Characteristics			
Island Age (Log_10_-transformed)	−0.494	0.019	22
Island Area (Log_10_-transformed)	0.454	0.034	22
Lizard Density (Log_10_-transformed)	−0.467	0.028	22
Percent Openness	0.331	0.132	22
Number of Predator Types	0.559	0.007	22

**Table 3:** Selection criteria for the sixteen mixed models constructed to explain variation in maximum sprint speed of Cycladic populations of *P. erhardii*. The output of the best model is boldfaced. All models included maximal sprint speed as the dependent variable, with sex, SVL, and body temperature as covariates. The right two columns give the results of adding island age to each model given in column one. Sprint speed, SVL, and island age were log_10_ transformed for analyses. The predator predictors were R = rats, SB = sand boas [*Eryx jaculus*], B = predatory birds, V = vipers [*Vipera ammodytes*], OS = other saurophagous Colubrid snakes, and M = mammals. Zero Predation is the presence of absence of any predator on the island, and “Sum of All Fears” uses the count of all the types of predators found on an island.

	**Base Model**		**Model plus Island Age**	
**Model Parameters**	**ΔAICc**	**Akaike Weight**	**ΔAICc**	**Akaike Weight**
Mammals	**0**	**0.228**	1.737	0.096
Mammals + Zero Predation	0.641	0.165	1.399	0.113
Zero Predation	1.357	0.116	2.896	0.054
Birds + Zero Predation	1.416	0.112	7.989	0.004
Sum of All Fears	3.231	0.045	6.654	0.008
Mammals + Birds	4.444	0.025	6.531	0.009
Birds	4.808	0.021	7.551	0.005
R+SB+V+B+OS+M	16.616	5.61 × 10^−5^	17.296	3.99 × 10^−5^

## Data Availability

The data are available from the corresponding author.
